# Impact of Inflammatory Bowel Disease on Outcomes After Coronary Interventions: A Systematic Review and Meta-Analysis

**DOI:** 10.7759/cureus.115696

**Published:** 2026-09-03

**Authors:** Sariyah Fatima, Uday Shree Akkala Shetty, Anastasia Postoev, Venkata Anirudh Chunchu, Sidra Jabeen, Sonalben Chaudhary, Rahman Hameed Mohammed Abdul, Adil Amin

**Affiliations:** 1 Medicine, Avicenna Medical College, Lahore, PAK; 2 Internal Medicine, Southern Regional Medical Center, Riverdale, GEO; 3 Internal Medicine, Caribbean Medical University, Willemstad, CUW; 4 Medicine, Avalon University School of Medicine, Willemstad, CUW; 5 Internal Medicine, Latin American School of Medicine (ELAM), Havana, CUB; 6 Internal Medicine, Zydus Sitapur Hospital, Sitapur, IND; 7 Gastroenterology and Hepatology, King's Mill Hospital, Sutton-in-Ashfield, GBR; 8 Cardiology, PNS Shifa, Karachi, PAK

**Keywords:** coronary artery bypass grafting, inflammatory bowel disease, major adverse cardiovascular events, meta-analysis, percutaneous coronary intervention

## Abstract

Inflammatory bowel disease (IBD) is associated with an elevated long-term cardiovascular risk through chronic systemic inflammation, yet whether it adversely affects outcomes following coronary revascularization remains uncertain. We conducted a systematic review and meta-analysis to quantify the association between IBD and clinical outcomes after percutaneous coronary intervention or coronary artery bypass grafting. MEDLINE, Embase, CENTRAL, Scopus, and Web of Science were searched from inception to June 20, 2026. Observational studies comparing patients with IBD with non-IBD controls undergoing coronary intervention and reporting at least one prespecified outcome were eligible. Five studies were included. Pooled analysis using a DerSimonian-Laird random-effects model demonstrated no statistically significant difference between IBD and non-IBD patients in the risk of major adverse cardiovascular events (MACE; risk ratio (RR) 1.01, 95% CI 0.84-1.21), all-cause mortality (RR 0.97, 95% CI 0.71-1.32), or stroke (RR 0.89, 95% CI 0.78-1.02). Heterogeneity was substantial for MACE and mortality (I² 97% and 94%, respectively) and moderate for stroke (I² 49%). Subgroup analysis revealed no significant difference in any outcome between Crohn’s disease and ulcerative colitis. The pooled null result, however, masks divergent signals across studies, with the sole long-term cohort reporting a 37% relative increase in MACE, in contrast to predominantly neutral or favorable findings from in-hospital administrative analyses. IBD does not appear to significantly worsen short-term outcomes after coronary intervention, although inflammation-driven risk may accrue over longer follow-up. Prospective studies incorporating disease activity, treatment exposure, and extended surveillance are warranted.

## Introduction and background

Inflammatory bowel disease (IBD), comprising Crohn’s disease (CD) and ulcerative colitis (UC), is a chronic immune-mediated disorder of the GI tract characterized by a relapsing-remitting course of intestinal inflammation [1]. Once considered a disease of the industrialized West, IBD has become a global health challenge, now affecting more than seven million people worldwide, with a prevalence forecast to approach 1% in several high-income regions within the next decade and incidence accelerating rapidly across newly industrialized nations [2,3]. As affected populations grow and age, an increasing number of patients with IBD will present with concurrent cardiovascular disease requiring intervention.

Chronic systemic inflammation, the hallmark of IBD, is a well-established driver of atherogenesis, promoting endothelial dysfunction, plaque instability, and a prothrombotic state [4]. Consistent with this mechanism, a large body of epidemiological evidence links IBD to an elevated long-term risk of ischemic heart disease, myocardial infarction, and other major adverse cardiovascular events (MACE), independent of, and often in the relative absence of, traditional cardiovascular risk factors [4,5]. These observations have positioned IBD as a cardiovascular risk-enhancing condition and have prompted interest in anti-inflammatory strategies for cardiovascular prevention [6].

Percutaneous coronary intervention (PCI) and coronary artery bypass grafting (CABG) are the cornerstones of revascularization for obstructive coronary artery disease. Whether the chronic inflammatory milieu of IBD adversely affects outcomes after these procedures, however, remains uncertain. The peri- and post-interventional period is a vulnerable window in which persistent low-grade inflammation, hypercoagulability, and heightened GI bleeding risk may plausibly increase rates of recurrent ischemia, in-stent restenosis, heart failure, and procedural complications [7,8]. Yet the available evidence is limited and conflicting. Several administrative database analyses have reported similar or even lower in-hospital mortality and MACE in patients with IBD undergoing PCI, potentially reflecting a favorable traditional risk-factor profile and younger age at presentation [8,9], whereas cohort studies incorporating longer-term follow-up have demonstrated a significantly increased risk of MACE, heart failure, and revascularization [7,10]. Differences in study design, outcome definitions, effect measures, and follow-up duration have contributed to this heterogeneity, and no quantitative synthesis focused specifically on outcomes after coronary intervention currently exists.

Clarifying whether IBD independently worsens outcomes after PCI or CABG has direct implications for preprocedural risk stratification, antithrombotic decision-making, and post-interventional surveillance in a rapidly expanding patient population. We therefore conducted a systematic review and meta-analysis to pool the available evidence and quantify the association between IBD and clinical outcomes, including mortality, MACE, and procedural complications following coronary interventions.

## Review

Methodology

This systematic review and meta-analysis was conducted in accordance with the PRISMA 2020 guidelines and the Meta-analysis of Observational Studies in Epidemiology (MOOSE) recommendations [11,12]. The protocol for this meta-analysis was registered with PROSPERO (CRD460254554819).

Search Strategy and Literature Search

We systematically searched MEDLINE (via PubMed), Embase, the Cochrane Central Register of Controlled Trials (CENTRAL), Scopus, and Web of Science from database inception to June 20, 2026, with no language restrictions. The search combined controlled vocabulary (MeSH and Emtree) and free-text terms for the two core concepts using Boolean operators, structured broadly as ("inflammatory bowel disease" OR "Crohn disease" OR "Crohn’s disease" OR "ulcerative colitis" OR IBD) AND ("percutaneous coronary intervention" OR PCI OR "coronary artery bypass" OR CABG OR "coronary revascularisation" OR "coronary revascularization" OR "coronary intervention" OR "coronary angioplasty" OR stent). The detailed search strategy is presented in Appendix A.

To identify additional eligible records, we hand-searched the reference lists of all included studies and relevant narrative and systematic reviews and screened conference abstracts and preprint repositories. Where a study was available only as a conference abstract but had since been published as a full manuscript based on the same dataset, only the full publication was retained to avoid duplication. Records were exported to reference management software and deduplicated. Two reviewers independently screened titles and abstracts, followed by full-text assessment of potentially eligible articles. Disagreements at either stage were resolved by discussion or, where consensus could not be reached, by adjudication from a third reviewer.

Eligibility Criteria

Studies were eligible if they met the following criteria, framed according to the Population, Exposure, Comparator, Outcome (PECO) structure. The population comprised adult patients (≥18 years) undergoing coronary intervention (PCI and/or CABG). The exposure was a diagnosis of IBD (CD, UC, or IBD unclassified). The comparator was patients without IBD undergoing the same intervention. Studies were required to report at least one outcome of interest with an effect estimate or sufficient raw data to permit its calculation.

We included observational study designs (cohort, case-control, and cross-sectional analyses, including administrative database studies) and any interventional studies meeting the above criteria. We excluded reviews, editorials, commentaries, case reports, case series without a comparator group, animal or in vitro studies, and studies in which outcomes for the coronary-intervention subgroup could not be disaggregated from a broader cardiovascular population. Where multiple studies reported on overlapping populations from the same data source and time period, the study with the most complete outcome data or largest sample was retained for the primary analysis.

Data Extraction

Two reviewers independently extracted data using a standardized, piloted extraction form, with discrepancies resolved by consensus. Extracted variables included first author, year of publication, country, study design, sample size (IBD and comparator groups), IBD subtype distribution, and type of coronary intervention.

The prespecified outcomes were MACE, all-cause mortality, and stroke. For each outcome, we extracted the most fully adjusted effect estimate reported with its corresponding 95% CI, together with the number of events and total participants in each group where available. Because the constituent components of MACE varied across studies, we recorded each study's specific MACE definition and planned to account for this heterogeneity in the analysis. When a study reported both crude and adjusted estimates, the adjusted (or propensity-matched) estimate was used preferentially. Where an effect estimate was not reported but raw event counts were available, we calculated the estimate directly.

Quality Assessment

Two reviewers independently assessed the methodological quality and risk of bias of each included study using the Newcastle-Ottawa Scale for observational studies, which evaluates three domains: selection of the study groups, comparability of groups (with particular attention to adjustment for traditional cardiovascular risk factors), and ascertainment of the outcome or exposure [13]. Studies scoring ≥7 of a possible 9 stars were considered high quality, 4-6 moderate, and <4 low. Disagreements were resolved by discussion or third-reviewer adjudication.

Analysis Plan

Effect estimates were pooled using a DerSimonian-Laird random-effects model, chosen a priori in anticipation of clinical and methodological heterogeneity across differing populations, data sources, and outcome definitions [14]. Because the included studies reported different effect measures, including risk ratios (RRs) and ORs from studies with in-hospital or short-term follow-up and hazard ratios (HRs) from studies with longer-term, time-to-event follow-up, a generic inverse-variance method was used to pool estimates on the log-relative-effect scale, rather than a model assuming a single, uniform effect measure.

For each outcome, RR with 95% CI will be presented and displayed in forest plots. Statistical heterogeneity was quantified using the I² statistic and the Cochran Q test, with I² values of approximately 25%, 50%, and 75% interpreted as low, moderate, and substantial heterogeneity, respectively. To explore sources of heterogeneity, we planned prespecified subgroup analyses by IBD subtype (CD vs UC). A two-sided p-value of <0.05 was considered statistically significant. All analyses were performed using Review Manager (RevMan; Cochrane, London, UK).

Results

Figure 1 presents the PRISMA flowchart of study selection. Overall, 634 studies were identified through online database searching. After removing duplicates, 568 studies were initially screened using their titles and abstracts. Of these, the full texts of 12 studies were obtained, and detailed screening was performed. Finally, five studies were included in this meta-analysis [7-9,15,16].

**Figure 1:**
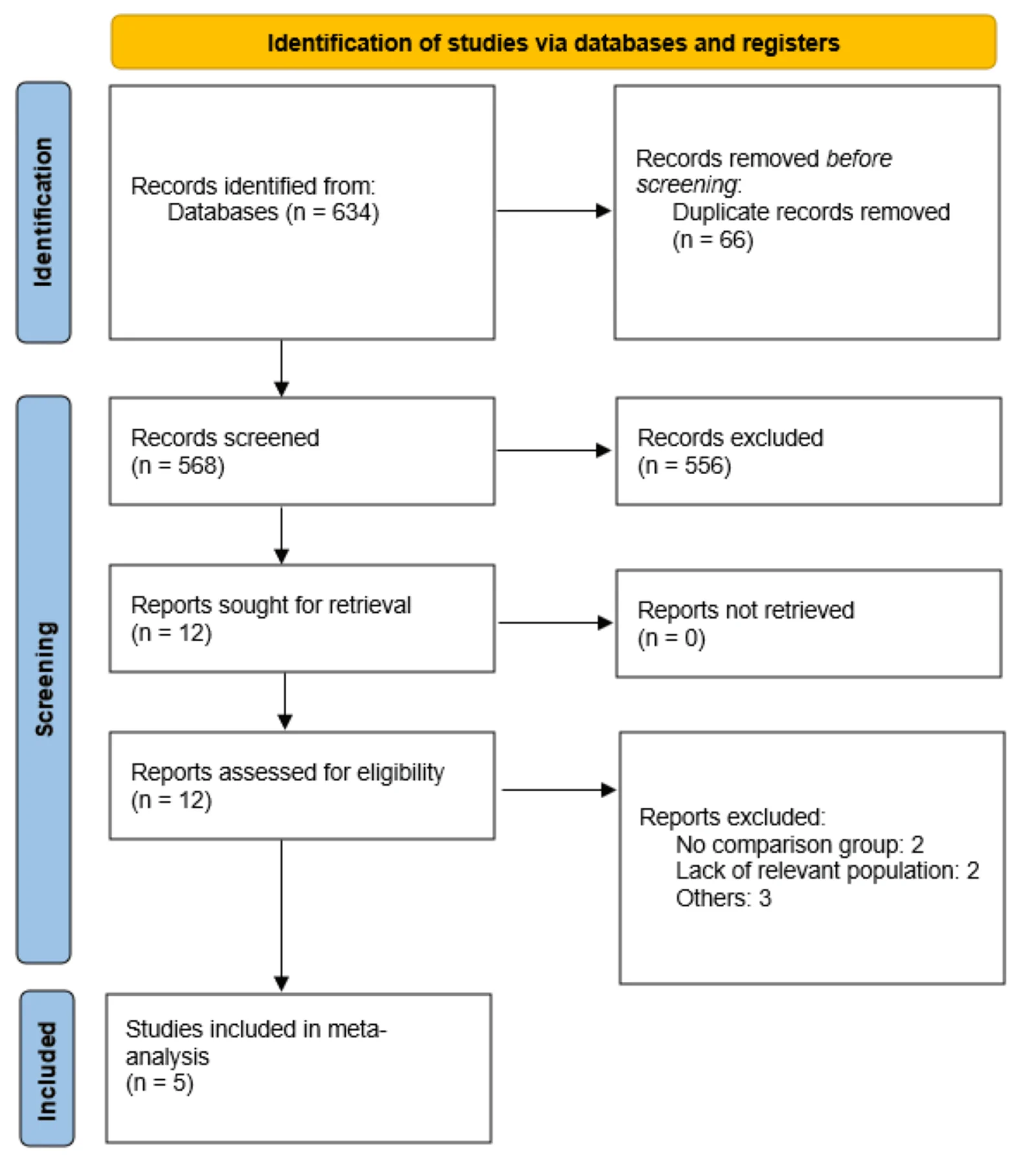
Study selection process.

Study Characteristics

Table 1 presents the characteristics of the included studies. Of the five studies, four compared patients with IBD with those without IBD. In one study [16], only comparative data for UC and CD were available. Of the five included studies, three were conducted in subjects with PCI only, while two studies included both PCI and CABG patients [9].

**Table 1:** Characteristics of included studies. CABG, coronary artery bypass grafting; CD, Crohn’s disease; IBD, inflammatory bowel disease; NIS, National Inpatient Sample; NR, not reported; NRD, National Readmission Database; PCI, percutaneous coronary intervention; UC, ulcerative colitis

Author (year)	Region	Study design	Subjects with IBD (n)	Follow-up duration	Males (n)	PCI (n)	CABG (n)
Ebrahimi et al. (2025) [7]	Switzerland	Nationwide propensity score-matched cohort	987	Median 3.5 years	720	813	174
Kobo et al. (2020) [8]	United States	Nationwide cohort (NIS), multivariable-adjusted	18,910	In-hospital	11,724	18,190	0
Bhagat et al. (2026) [9]	United States	Nationwide propensity score-matched cohort (NRD)	11,499	In-hospital (≤11 months)	7,425	NR	NR
Jain et al. (2026) [15]	United States	Multi-institutional propensity score-matched cohort (TriNetX)	4,578	12 months	2,875	4,578	0
Savren Lotker et al. (2024) [16]	Israel	Single-center retrospective matched cohort	63	Long-term (mean ≈5 years)	44	63	0

Risk of Bias Assessment

Table 2 presents the quality assessment of the included studies. Three studies scored 8-9/9, reflecting adequate cohort selection, adjustment for confounders, and outcome ascertainment. Savren Lotker et al. [16] scored lowest (6/9), reflecting its smaller single-center sample and more limited covariate adjustment, warranting cautious interpretation of its contribution to the pooled estimates.

**Table 2:** Quality assessment of included studies.

Author (year)	Selection (/4)	Comparability (/2)	Outcome (/3)	Total (/9)
Ebrahimi et al. (2025) [7]	4	2	3	9
Kobo et al. (2020) [8]	4	2	2	8
Bhagat et al. (2026) [9]	4	2	2	8
Jain et al. (2026) [15]	4	2	3	9
Savren Lotker et al. (2024) [16]	3	1	2	6

Meta-analysis of outcomes

MACE

Figure 2 presents the forest plot comparing the risk of MACE between patients with IBD and non-IBD controls. The pooled analysis demonstrated no significant difference in the risk of MACE between the two groups (RR: 1.01, 95% CI: 0.84-1.21). A high degree of heterogeneity was observed across the included studies (I² = 97%).

**Figure 2:**
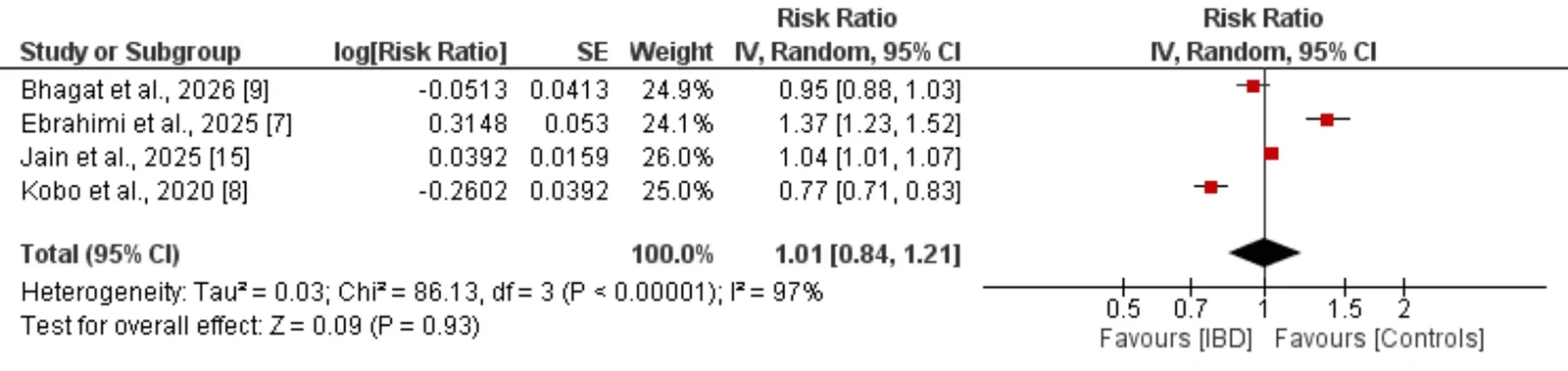
Comparison of MACE between two groups. IBD, inflammatory bowel disease; MACE, major adverse cardiovascular events [7-9,15]

Mortality

Figure 3 illustrates the forest plot comparing all-cause mortality between patients with IBD and non-IBD controls. The pooled results indicated no significant difference in mortality risk between the two groups (RR: 0.97, 95% CI: 0.71-1.32). Considerable heterogeneity was identified among the studies (I² = 94%).

**Figure 3:**
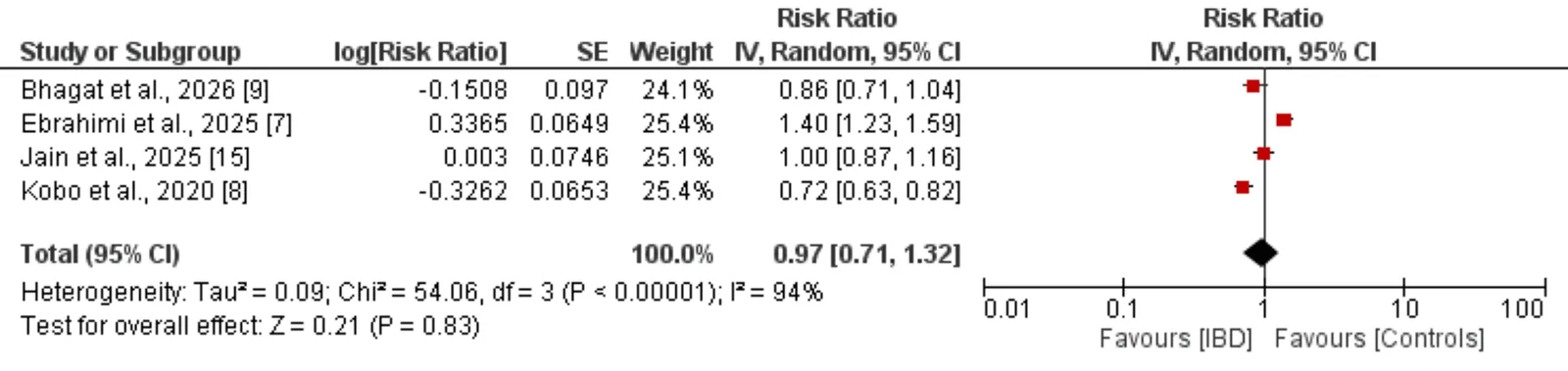
Comparison of mortality between two groups. IBD, inflammatory bowel disease [7-9,15]

Stroke

Figure 4 presents the forest plot comparing the risk of stroke between patients with IBD and non-IBD controls. The pooled analysis showed no statistically significant difference in stroke risk between the groups (RR: 0.89, 95% CI: 0.78-1.02). Moderate heterogeneity was observed across the included studies (I² = 49%).

**Figure 4:**
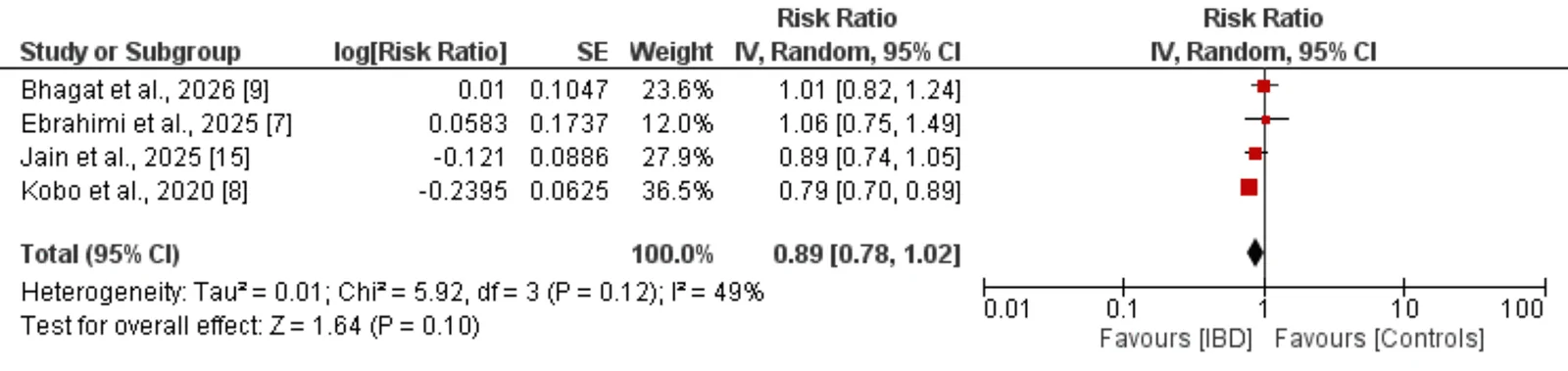
Comparison of stroke between two groups. IBD, inflammatory bowel disease [7-9,15]

Subgroup analysis: CD vs UC

Table 3 summarizes the subgroup analysis comparing the risks of MACE, mortality, and stroke between patients with CD and UC. The pooled estimates revealed no statistically significant differences between the two IBD subtypes for any of the assessed outcomes. However, these findings should be interpreted cautiously due to the limited number of studies included in the subgroup analyses.

**Table 3:** Subgroup analysis. MACE, major adverse cardiovascular events; RR, risk ratio

Outcomes	RR (95% CI)	I²
MACE	0.95 (0.86-1.05)	26%
All-cause mortality	1.35 (0.93-1.95)	66%
Stroke	1.00 (0.65-1.54)	84%

Discussion

In this systematic review and meta-analysis of five studies comprising a large aggregate population of patients undergoing coronary intervention, we found that IBD was not associated with a statistically significant difference in the risk of MACE (RR 1.01, 95% CI 0.84-1.21), all-cause mortality (RR 0.97, 95% CI 0.71-1.32), or stroke (RR 0.89, 95% CI 0.78-1.02) compared with non-IBD comparators. Subgroup analysis comparing CD with UC likewise showed no significant difference across the three outcomes. These pooled null findings should, however, be interpreted in the context of the marked heterogeneity described below, which reflects real differences in study design rather than a straightforward absence of effect.

Our neutral pooled result is broadly concordant with the wider body of work examining IBD in the setting of acute coronary presentations, most of which similarly fails to demonstrate excess short-term hard cardiovascular outcomes. In the largest such analysis, Pemmasani et al. examined 24,220 acute coronary syndrome hospitalizations among patients with IBD in the US National Inpatient Sample and found that in-hospital mortality was actually lower in the IBD cohort (3.9% vs 5.3%; adjusted OR 0.81, 95% CI 0.69-0.96), with no difference between UC and CD, directly paralleling the neutral subtype comparison in our analysis [17]. Similarly, Sinh et al. reported that IBD did not affect mortality after acute myocardial infarction, although it prolonged hospitalization [18]. Notably, Pemmasani et al. identified GI bleeding, coagulopathy, and weight loss as strong independent predictors of death in IBD patients, echoing the elevated bleeding and GI complication signal seen in Kobo et al. and Bhagat et al. and reinforcing our interpretation that the excess risk in this population lies predominantly outside the conventional MACE composite [8,9,16,18].

The pattern we observed also mirrors findings in other chronic inflammatory conditions undergoing coronary intervention, suggesting that a null or paradoxically favorable periprocedural signal is not unique to IBD. Analyses of patients with rheumatoid arthritis and systemic lupus erythematosus undergoing PCI have produced mixed results, with some demonstrating worse long-term outcomes such as restenosis and repeat revascularization, yet in-hospital administrative analyses frequently show comparable or lower short-term event rates, attributable to younger age at presentation and a lower prevalence of traditional risk factors [19]. This consistency across autoimmune diseases supports the hypothesis that the adverse cardiovascular consequences of chronic systemic inflammation manifest cumulatively over extended follow-up rather than during the index procedure and that cross-sectional in-hospital endpoints, which dominate our pooled dataset, are poorly suited to capturing them.

Finally, our null post-interventional findings stand in apparent contrast to the substantial literature establishing IBD as a risk factor for incident cardiovascular disease. Meta-analyses and large cohort studies have consistently reported elevated risks of myocardial infarction, ischemic heart disease, and acute coronary syndrome in the general IBD population, with a Taiwanese nationwide cohort demonstrating an 87% higher incidence of ACS (adjusted HR 1.72, 95% CI 1.53-1.94) and the relative risk being greatest in younger patients [4,20]. This discordance is instructive: it indicates that the question of whether IBD causes cardiovascular disease is distinct from whether IBD worsens outcomes once coronary disease requires intervention. The former is dominated by the long-term atherogenic effect of inflammation, whereas the latter is shaped by competing forces, namely, a favorable baseline risk-factor profile and younger age on one hand, and heightened bleeding and GI vulnerability on the other, that may cancel out at the level of the MACE composite. Reconciling these two literatures will require prospective studies that track patients with IBD from intervention through long-term follow-up while accounting for disease activity and treatment exposure.

The subtype analysis showed no significant difference between CD and UC for any outcome, consistent with the individual-study findings of Ebrahimi et al., who reported near-identical MACE hazards for the two subtypes [7]. However, Kobo et al. observed substantially lower adjusted mortality in UC than in CD, and the small number of studies contributing subtype-specific data limits the power and reliability of this comparison; these results should therefore be regarded as exploratory [8].

Limitations

The substantial heterogeneity observed for the primary outcome (I² = 97%) reflects genuine methodological diversity across the included studies and is the most consequential limitation of this review. Most importantly, the included studies used nonuniform effect measures, combining in-hospital ORs [8,9] with longer-term HRs and RRs [7,15,16]. These were pooled using a generic inverse-variance approach under the assumption of approximate equivalence between RR, OR, and HR, which is a recognized but imperfect approach when outcomes are not rare and follow-up periods differ substantially, as they do here (in-hospital only vs a mean of five years). We consider this the most likely driver, rather than a mere coincidence, of the extreme heterogeneity observed for MACE and mortality (I² = 97% and 94%, respectively), and the pooled estimates for these outcomes should therefore be interpreted as hypothesis-generating rather than as a precise unified effect size. We were not able to perform sensitivity or subgroup analyses stratified by follow-up duration or effect measure, given the small number of eligible studies (k = 5).

Beyond effect-measure heterogeneity, MACE composites were also inconsistently defined across studies, incorporating differing component events, and confounding adjustment varied: some studies applied propensity score matching [7,9,15], while others used multivariable adjustment [8] or, given a small single-center sample, limited covariate control [16]. Data sources, calendar periods, and populations also differed, spanning national registries and multi-institutional networks across Switzerland, the United States, and Israel.

Further limitations include the small number of eligible studies overall, which limits statistical power and precluded formal assessment of publication bias through funnel-plot asymmetry; the exclusively observational design of all included studies, most of which relied on administrative coding subject to misclassification and lacked data on IBD disease activity, remission status, biologic and corticosteroid exposure, and procedural details such as stent type, any of which may confound the association; and the predominantly US and Western European origin of included cohorts, limiting generalizability. Finally, most included studies assessed only patients undergoing PCI; only two also evaluated CABG.

Future Implications

Future research should prioritize long-term follow-up, as most included studies [8,9] captured only in-hospital outcomes, whereas the sole long-term cohort [7] showed elevated MACE, suggesting that inflammation-driven risk accrues over years. Standardized, guideline-endorsed MACE definitions with separately reported components are needed to reduce the heterogeneity (I² = 97%) that limited pooling. Critically, no study accounted for IBD disease activity or treatment exposure; future work should stratify outcomes by flare status, corticosteroid vs biologic therapy, and inflammatory biomarkers. Studies should also incorporate bleeding and GI endpoints, adequately powered CD-vs-ulcerative-colitis comparisons, and more ethnically diverse, prospectively followed populations to establish true post-interventional risk.

## Conclusions

This systematic review and meta-analysis found no statistically significant association between IBD and the risk of MACE, all-cause mortality, or stroke following coronary intervention, with no meaningful difference between CD and UC. However, these pooled estimates were limited by substantial heterogeneity arising from differences in study design, follow-up duration, outcome definitions, and confounding adjustment and should therefore be regarded as hypothesis-generating rather than conclusive. The divergent signals across individual studies, particularly between short-term and long-term data, underscore the need for well-designed prospective studies with standardized outcomes, longer follow-up, and adjustment for disease activity and treatment exposure.

## Appendices

Appendix A: Search strategy

PubMed: ("Inflammatory Bowel Diseases"[MeSH] OR "Crohn Disease"[MeSH] OR "Colitis, Ulcerative"[MeSH] OR "inflammatory bowel disease"[tiab] OR "Crohn disease"[tiab] OR "Crohn's disease"[tiab] OR "ulcerative colitis"[tiab] OR IBD[tiab]) AND ("Percutaneous Coronary Intervention"[MeSH] OR "Coronary Artery Bypass"[MeSH] OR "percutaneous coronary intervention"[tiab] OR PCI[tiab] OR "coronary artery bypass"[tiab] OR CABG[tiab] OR "coronary revascularisation"[tiab] OR "coronary revascularization"[tiab] OR "coronary intervention"[tiab] OR "coronary angioplasty"[tiab] OR stent[tiab] OR stenting[tiab])

EMBASE: ('inflammatory bowel disease'/exp OR 'crohn disease'/exp OR 'ulcerative colitis'/exp OR 'inflammatory bowel disease':ti,ab OR 'crohn disease':ti,ab OR 'crohn''s disease':ti,ab OR 'ulcerative colitis':ti,ab OR IBD:ti,ab) AND ('percutaneous coronary intervention'/exp OR 'coronary artery bypass graft'/exp OR 'percutaneous coronary intervention':ti,ab OR PCI:ti,ab OR 'coronary artery bypass':ti,ab OR CABG:ti,ab OR 'coronary revascularisation':ti,ab OR 'coronary revascularization':ti,ab OR 'coronary intervention':ti,ab OR 'coronary angioplasty':ti,ab OR stent:ti,ab OR stenting:ti,ab)

CENTRAL:#1 MeSH descriptor: [Inflammatory Bowel Diseases] explode all trees #2 MeSH descriptor: [Crohn Disease] explode all trees #3 MeSH descriptor: [Colitis, Ulcerative] explode all trees #4 ("inflammatory bowel disease" OR "Crohn disease" OR "Crohn's disease" OR "ulcerative colitis" OR IBD):ti,ab,kw #5 #1 OR #2 OR #3 OR #4 #6 MeSH descriptor: [Percutaneous Coronary Intervention] explode all trees #7 MeSH descriptor: [Coronary Artery Bypass] explode all trees #8 ("percutaneous coronary intervention" OR PCI OR "coronary artery bypass" OR CABG OR "coronary revascularisation" OR "coronary revascularization" OR "coronary intervention" OR "coronary angioplasty" OR stent*):ti,ab,kw #9 #6 OR #7 OR #8 #10 #5 AND #9

Scopus: ( TITLE-ABS-KEY ( "inflammatory bowel disease" OR "Crohn disease" OR "Crohn's disease" OR "ulcerative colitis" OR IBD ) ) AND ( TITLE-ABS-KEY ( "percutaneous coronary intervention" OR PCI OR "coronary artery bypass" OR CABG OR "coronary revascularisation" OR "coronary revascularization" OR "coronary intervention" OR "coronary angioplasty" OR stent* ) )

Web of Science: TS=("inflammatory bowel disease" OR "Crohn disease" OR "Crohn's disease" OR "ulcerative colitis" OR IBD) AND TS=("percutaneous coronary intervention" OR PCI OR "coronary artery bypass" OR CABG OR "coronary revascularisation" OR "coronary revascularization" OR "coronary intervention" OR "coronary angioplasty" OR stent*)
